# Social Category Formation Is Induced by Cues of Sharing Knowledge in Young Children

**DOI:** 10.1371/journal.pone.0101680

**Published:** 2014-07-11

**Authors:** Katalin Oláh, Fruzsina Elekes, Gábor Bródy, Ildikó Király

**Affiliations:** 1 Department of Cognitive Psychology, Eötvös Loránd University, Budapest, Hungary; 2 Institute of Cognitive Neuroscience and Psychology, Hungarian Academy of Sciences, Budapest, Hungary; The University of Chicago, United States of America

## Abstract

Previous research has shown that human infants and young children are sensitive to the boundaries of certain social groups, which supports the idea that the capacity to represent social categories constitutes a fundamental characteristic of the human cognitive system. However, the function this capacity serves is still debated. We propose that during social categorization the human mind aims at mapping out social groups defined by a certain set of shared knowledge. An eye-tracking paradigm was designed to test whether two-year-old children differentially associate conventional versus non-conventional tool use with language-use, reflecting an organization of information that is induced by cues of shared knowledge. Children first watched videos depicting a male model perform goal-directed actions either in a conventional or in a non-conventional way. In the test phase children were presented with photographs taken of the model and of a similarly aged unfamiliar person while listening to a foreign (Experiment 1) or a native language (Experiment 2) text. Upon hearing the foreign utterance children looked at the model first if he had been seen to act in an unconventional way during familiarization. In contrast, children looked at the other person if the model had performed conventional tool use actions. No such differences were found in case of the native language. The results suggest that children take the conventionality of behavior into account in forming representations about a person, and they generalize to other qualities of the person based on this information.

## Introduction

Human societies are unique among species in that our form of living entails a level of interdependence between conspecifics that cannot be found in any other animal species. Humans form alliances with other people for different purposes every day, during which they engage in a wide variety of joint actions and collaboration towards goals, such as creating artifacts, working towards scientific discoveries or – even – doing sports together [1], [2]. Group-living constitutes a fundamental characteristic of the human race and group-affiliations can influence various aspects of our lives to a great extent. Therefore the ability to form cognitive representations of human groups can be seen as an evolutionary adaptive capacity of the human brain.

The phenomenon that adults have a propensity to think of fellow humans as belonging to groups has been well documented in the social psychology literature (see [3] for a review) and there is also ample evidence suggesting that even infants are able to perceive the boundaries of certain social categories. Studies conducted in the field of developmental psychology as well as cultural anthropology have demonstrated that young children or even infants are sensitive to social categories such as age [4], sex [5]–[9], race [10]–[14]. It has also been demonstrated that children as young as 2.5 years of age – similarly to adults [15] – are willing to accept even arbitrary cues of group membership when forming expectations about a person's behavior [16], however other studies suggest that some classifications have more limited power in guiding young children's behavior towards people [17].

More recently, in a new line of research, Kinzler and colleagues have shown that spoken language occupies a prominent role in young children's representations of social categories [18]–[20]. Their findings also indicate that this distinction is more privileged in children's eyes than some other, such as race [21], which suggests that language taps into some of the more fundamental processes underlying the propensity of category-based thinking. One of the most important findings of these experiments is that the accent with which a person speaks has a stronger effect on 5-year-olds attitude toward that person than their ethnicity.

Despite the great and long interest and the social relevance of the question of social categorization, the cognitive basis and the function of this process is still somewhat under-explained. Theories in social psychology have provided numerous accounts of how affiliating with a group may benefit an individual in – for example – boosting their self-esteem [15], [22]; justifying a bigger share of resources [23]–[24], etc. These accounts generally tend to emphasize the competition between different social groups and assume an antagonist relationship between them. Theorists from various fields of cognitive sciences tend to grasp a different aspect of the question and offer other explanations with respect to the function of group-based thinking. Sperber and Hirschfeld [25] for example argue that the human mind has evolved a special domain to reason about social kinds in order to make sense of the extremely complicated structure of human societies. Similarly, Cosmides, Tooby, and Kurzban [26] propose that the primary function of representing social categories is to map out potential coalitional partners.

An adaptive system that is responsible for representing social groups must correspond to the core characteristics and advantages of group living. Forming short or long term bonds with other people enables us to create the uniquely complex and developed cultural niche that we live in. These products of human cooperation are also unique in that they can be preserved over time by the help of a specially evolved communicational system that allows for the efficient transmission of knowledge from one generation to the other [27]–[28]. As a result, each human culture is characterized by a vast body of shared knowledge that contains generic information about the world (such as “snow is cold”) as well as a set of mostly arbitrary cultural norms. The very nature of these cultural norms is that while they are in great part arbitrary, acquiring knowledge of the norms specific to a certain culture is crucial in managing everyday life and in interacting with others (imagine driving on the wrong side of the road!).

We propose that the relevance of representing social groups is that it enables us to find the boundaries of culturally shared knowledge. Spoken language is obviously a part of this and can serve as a perfect indication of cultural group membership from the earliest periods of our lives as even 2-day-old infants are sensitive to language [29]. However, conducting successful interactions with others not only requires a commonly spoken tongue, but also knowledge of other social norms and conventions. Therefore, we propose that the basis of social categorization may be culturally shared knowledge in general. This notion is plausible if we consider that this distinction is genuinely meaningful and helpful in guiding behavior, whereas other distinctions, such as one based on skin-color, are in themselves empty categories. That is not to say that in certain situations some features cannot correlate with each other (see [30] for how perceptual markers of ethnicity can become associated with fundamentally norm-based social groups). However, characteristics such as race are not deterministic in this respect.

To directly investigate the question whether children are indeed sensitive to the boundaries of culturally shared knowledge, we designed two experiments where we sought to test whether children form similar representations of people based on other cues of sharing knowledge (using tools in a conventional or a non-conventional way) as they do based on language. The conventionality of tool-using behavior is an adequate index of sharing cultural knowledge as artifact functions have an inherent cultural aspect. It has been proposed that the differentiation of tools was a result of recursive tool making practices. In consequence, function knowledge includes cognitively opaque properties that could only be understood through cultural practices and culturally evolved teaching situations. Csibra and Gergely argue that communication of generic knowledge was selected as a consequence of the learnability problem induced by cognitively opaque contents in tool making practices [27], [28]. Our hypothesis was that distinct cues -potentially of shared knowledge- induce an organized representation of social categories. Children were first familiarized with videos depicting a model that performed either conventional or non-conventional tool-use actions. Then, we tested whether children differentially associated a foreign language (Experiment 1) or their mother tongue (Experiment 2) to the model or a stranger based on the kind of tool-use the model had previously performed. We expected to find a difference between conditions in Experiment 1, but not in Experiment 2 as we hypothesized that the strong familiarity effect of hearing their native language would overshadow other effects.

## Experiment 1

### Method

#### Participants

Thirty (13 girls; mean age: 24.37 months, SD: 2.27 months) monolingual children between the age of 20 and 28 months participated in the study, of whom 15 were assigned in the Conventional condition and 15 in the Non-conventional condition. Participants were selected from a database of volunteer families that had previously applied for participation. Children were excluded from participation if there was at least one person in their immediate family whose native tongue was not Hungarian. An additional seven children were tested, but later excluded from the sample due to experimenter error (2); the eye-tracker did not return any data for the given measure and behavior could not be coded by visual observation (2), children could not be calibrated properly (2) and child's parents turned out to be of two different cultures (1).

#### Ethic statement

The experiment was conducted with the approval of the Ethical Committee of the Faculty of Education and Psychology, Eötvös Loránd University. Parents of the children signed informed consent prior to participation.

#### Equipment

For video stimuli presentation and data collection a Tobii T60XL eye-tracker was used with the TobiiStudio 3.2 software. The screen's size was 52×32 cm and 1920×1200 pixels. We used a five point calibration throughout the experiment. Children who were included in the final sample provided at least 80% valid eye-tracking data.

#### Video stimuli

For the familiarization phase, three videos were created for each condition (Conventional and Non-conventional). In each of the videos, a male model performed a goal-directed action with a chosen tool. The three videos depicted three different tool-using actions. The setup in the video always included two visible goals (e.g. a plate of food and messy hair) and two possible tools to bring the goals about (e.g. a fork and a brush). In all the videos the model first non-verbally, but explicitly demonstrated his goal by reaching for one of the goal-objects. Then he grabbed one of the tools, examined and then rejected it (shaking his head). After that he grabbed the other tool, nodded on examining it, and then used the tool to bring the goal about. After the goal had been attained, he expressed satisfaction by nodding at the outcome. Importantly, in both conditions all the goals and possible tools were familiar to the children, but in the Non-conventional condition the associations between tools and goals were unfamiliar (e.g. using a fork to brush his hair), while in the Conventional condition associations were always familiar (e.g. using a fork to eat food). The length of the videos varied between 16 and 20 seconds. For detailed description of the videos see the Appendix. Note that in both the Conventional and Non-conventional condition, the tool that was chosen by the model could efficiently bring about the outcome. Moreover, in both conditions, the model performed the action with equal confidence, and without communicating with the participant in any way (he did not look into camera, did not smile, wave, etc.). Thus, the familiarization events only differed in their level of conventionality.

#### Procedure

On arriving to the laboratory, children had some time to explore the room and get comfortable in the company of two experimenters, while parents were briefed about the experiment and signed informed consent. After this, one of the experimenters escorted the child and their parent into the testing room and seated them in a chair in front of the monitor of the eye-tracker (at a distance of approx. 60–70 cm) with the child sitting on the parent's lap. The experimenter assisted with calibration, but left the room once it was finished. Before the stimuli began, the model entered the room and started manipulating the computer without looking at or talking to the children. This element of the procedure was added in order to avoid the possibility that in-group and out-group effects were weakened by the fact that it was the in-group experimenter that directed children's attention to the stimuli. After the model had also left the room, children were presented with the three familiarization videos. Children always saw either three Conventional on three Non-conventional videos, depending on condition.

The familiarization phase was immediately followed by the test in which two photographs appeared side-by-side on the screen. The pictures were 21×11 cm and were positioned on the left and the right side of the screen with a 10×11 cm line in the middle, separating them. One picture depicted the model, while the other photo was taken of another young man matched in age. After 6 seconds had elapsed the voice of a man was heard from the speakers, who spoke in Swedish for 14 seconds.

#### Data analysis

Four groups of area of interest were created for analyses combining two factors: who was the target person (model or the other person) and time window (before the onset of the audio stimulus and after). The cut-off point was set at eight seconds, which marks the end of the first utterance; therefore by this point children already had the opportunity to judge the familiarity of the language. The length of the *before* time window was 8 seconds, while the *after* time window lasted for 12 seconds.

To test whether children differentially associated the foreign language to the two men in the test phase based on experimental condition, we analyzed the direction of their first gaze on hearing the foreign language. This measure was introduced in order to detect the potentially organized information seeking by children: if conventionality of tool use induces social categorization based on shared knowledge, children could use this information to integrate the novel stimulus as well. Children were expected to associate foreign language use with the model only after non-conventional tool use, since both cues point to the model's possessing different cultural knowledge than the participant - thus children would look first at the model. On the other hand, in case of conventional tool use, children were hypothesized to search for the source of the foreign language utterance by looking first at the novel human face, since there was a mismatch between the social categories induced by the two different cues.

We also analyzed total visit duration in both time windows to explore any possible general preference towards either of the models. In addition we analyzed total visit durations for the familiarization videos in order to exclude the possibility that children were more attentive in the Non-conventional condition due to the surprising behavior exhibited by the model.

#### Results

An alpha level of .05 was used for all statistical tests. Sex and age were first always entered into the analyses, but were not significant in any case and were therefore removed from all models.

An independent samples T-test was performed on the percentage of looking at the three familiarization videos with condition as a between subject variable. The analyses revealed a marginal effect of condition (*t*(29) = 1.99, *p* = 0.057), showing that children attended to the videos slightly more in the Conventional condition (Mean percentage of looking in the Conventional and the Non-conventional condition: 98 and 94 percent, respectively).

**Figure 1:**
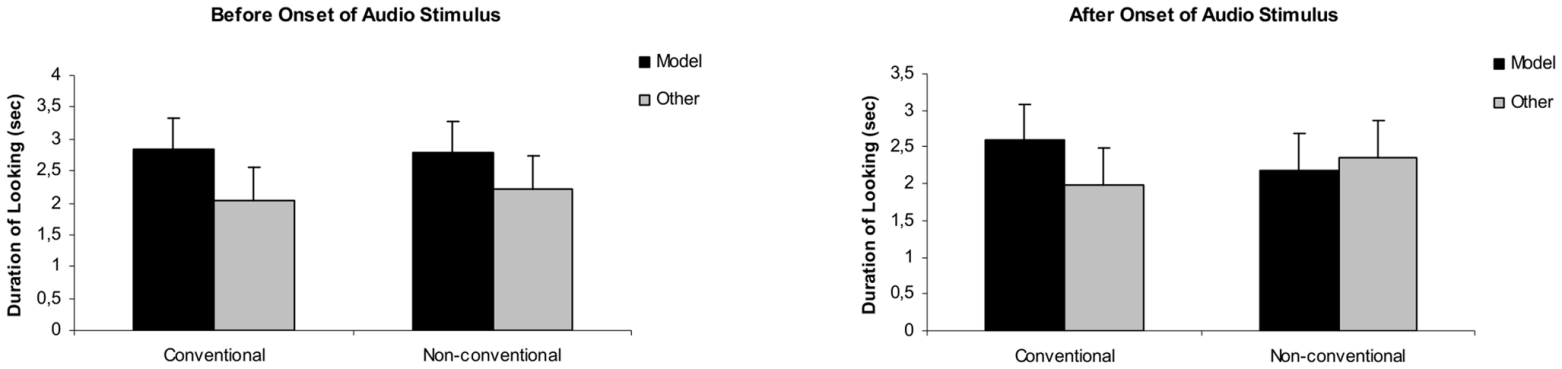
Total looking times in the test phase of Experiment 1. The duration of overall looking times at the two photographs (depicting the model and the other person) in the Conventional and Non-conventional condition, presented separately for the periods *before* and *after* the onset of the foreign language stimulus.

Repeated measures GLM analyses on the total visit durations (factors: target person [model vs. other], condition [Conventional vs. Non-conventional]) were performed separately for the *before* and the *after* time window in the test phase, which revealed a general preference towards the model (*F*(1,28) = 13.36, *p* = 0.001) *before* the onset of the audio stimulus (Mean looking times: Conventional condition/model: 2.84 sec; Conventional condition/other: 2.051sec; Non conventional condition/model: 2.79 sec; Non-conventional condition/other: 2.23 sec). This preference disappeared in the *after* time window (Mean looking times: Conventional condition/model: 2.59 sec; Conventional condition/other: 1.99 sec; Non-conventional/model: 2.19 sec; Non-conventional/other: 2.36 sec). See also Figure 1.

**Figure 2:**
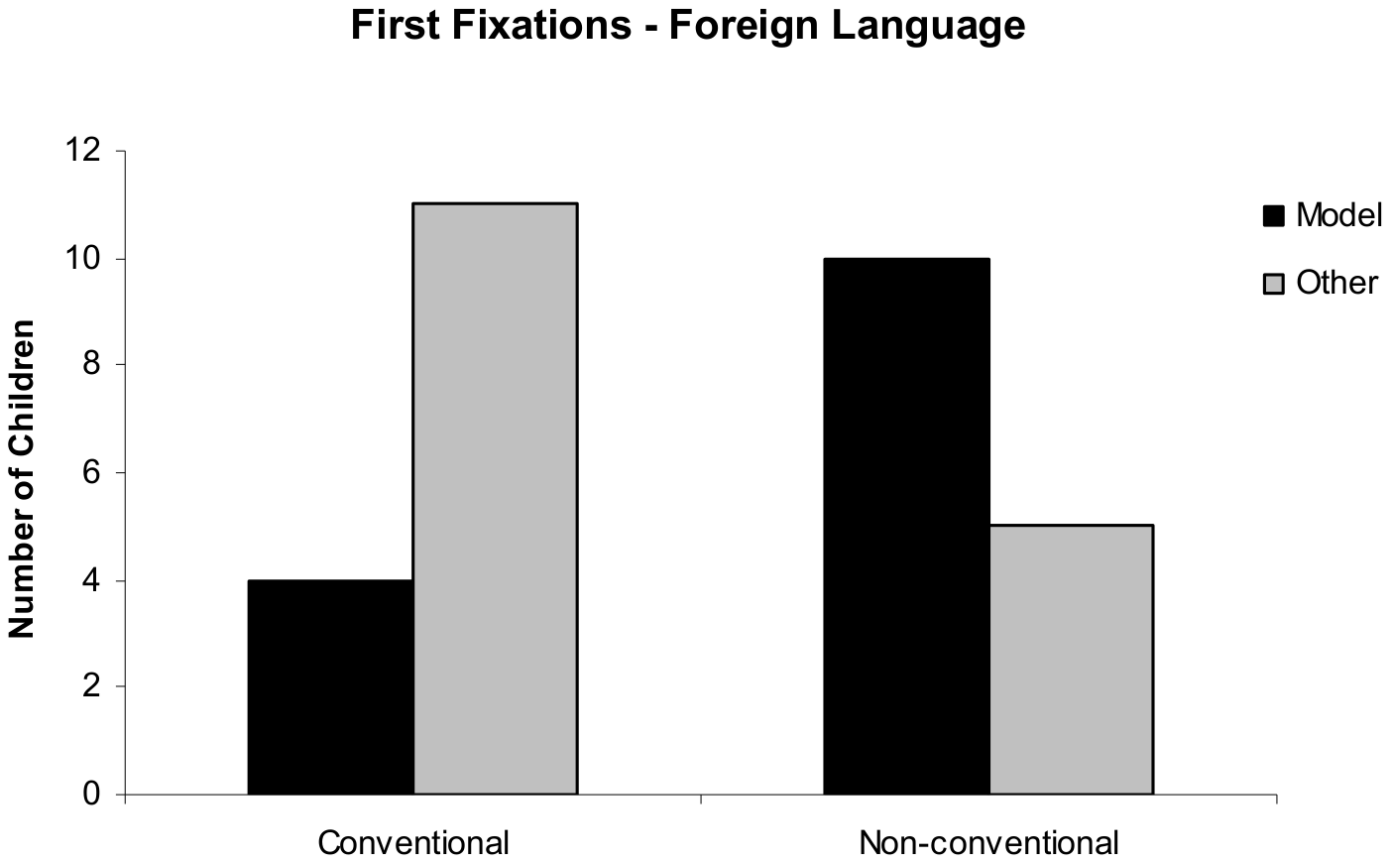
Distribution of first fixations after the onset of the audio stimulus in Experiment 1. Number of children looking first at the model and at the other person in the Conventional and the Non-conventional condition after hearing the foreign language text.

Crucially, analyzing the directions of the first fixations we found a significant effect of condition in the *after* time window (χ^2^ = 4.821, *p* = 0.028) showing that after the onset of the foreign language text (after time window), children were more likely to look at the model in the Non-conventional condition (10 out of 15 children), whereas the majority of children fixated on the other person first in the Conventional condition (11 out of 15 children). Results are depicted in Figure 2. No significant effect of condition was found *before* the audio stimulus (χ^2^ = 2.4, *p* = 0.12).

## Experiment 2

Experiment 2 was designed in order to test whether similar results could be obtained using the children's native language instead of a foreign language. Since first fixations can be regarded as part of an information seeking process after a certain stimulus, we expected it to be more indicative of children's cognitive processes after events that violate their expectations. Due to the fact that hearing people speak in our native tongue is such a strong part of our every-day experiences, we hypothesized that it would not elicit any specific cognitive processing, leading to a random pattern of first fixations in the two conditions. However, this study provides important additional information for interpreting the above described results and exploring the validity of the hypothesis that cues of shared knowledge may play a part in forming representations of social groups.

### Method

#### Participants

Twenty-six (10 girls, mean age: 24.32 month, SD: 2.28) monolingual children participated in the study with equal number of children assigned in the two conditions (Conventional and Non-conventional). The criterion for participation was the same as in Experiment 1. An additional three children were excluded from the sample due to inattentiveness.

#### Materials and procedure

The applied stimuli and the procedure were identical to the ones used in Experiment 1 with the exception that during the test phase, the Swedish audio text was replaced by a Hungarian (native) text. The length of the audio stimulus and the duration of the first utterance were matched to those in Experiment 1.

#### Results

An alpha level of .05 was used for all statistical tests. Age and sex were first entered into all of the models used in the analyses, but were later removed as they were not significant in any of the cases.

Pair-wise analysis of the percentages of the aggregated looking times during the three familiarization videos revealed a significant effect of condition, with children looking overall longer in the Conventional condition (*d*(25) = 3.08, *p* = 0.009). However, the difference between looking times was relatively small with a mean of 99 percent in the Conventional condition and 95 percent in the Non-conventional condition.

**Figure 3:**
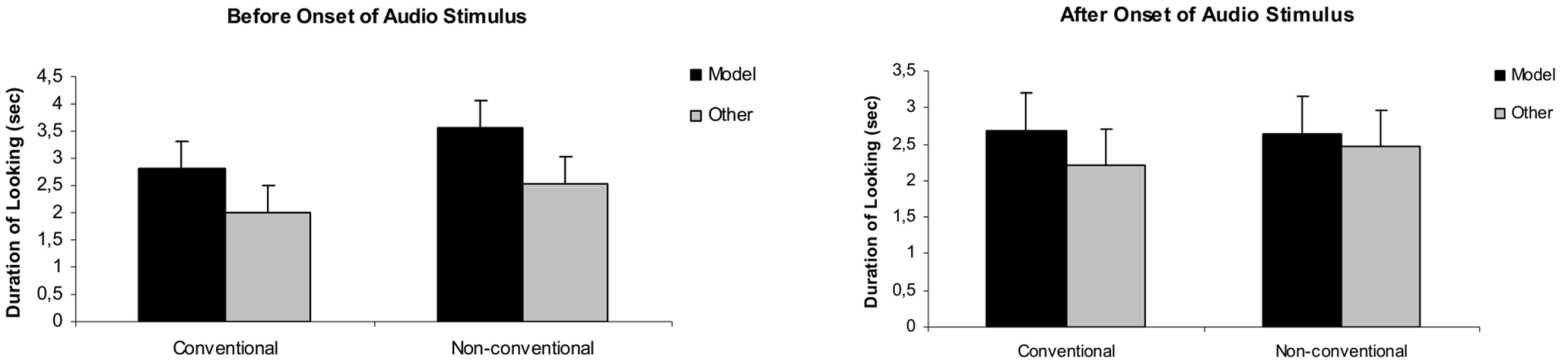
Looking times in the test phase of Experiment 2. The duration of overall looking times at the two photographs (depicting the model and the other person) in the Conventional and Non-conventional condition, presented separately for the periods *before* and *after* the onset of the native language stimulus.

A GLM analysis on the total visit durations in the test phase yielded a significant effect of condition (*F*(1, 24) = 24.34, *p*<0.001) and target person (*F*(1, 24) = 4.34, *p* = 0.048) in the *before* time window. Children spent more time looking at the model than at the other person and they looked longer in the Non-conventional than the Conventional condition (Conventional/model: 2.82 sec; Conventional/other: 1.99 sec; Non-conventional/model: 3.55 sec; Non-conventional/other: 2.53 sec). No effects were found in the *after* time-window (Conventional condition/model: 2.69 sec; Conventional condition/other: 2.21 sec; Non-conventional condition/model2.65 sec; Non-conventional condition/other: 2.47 sec, see also Figure 3).

**Figure 4:**
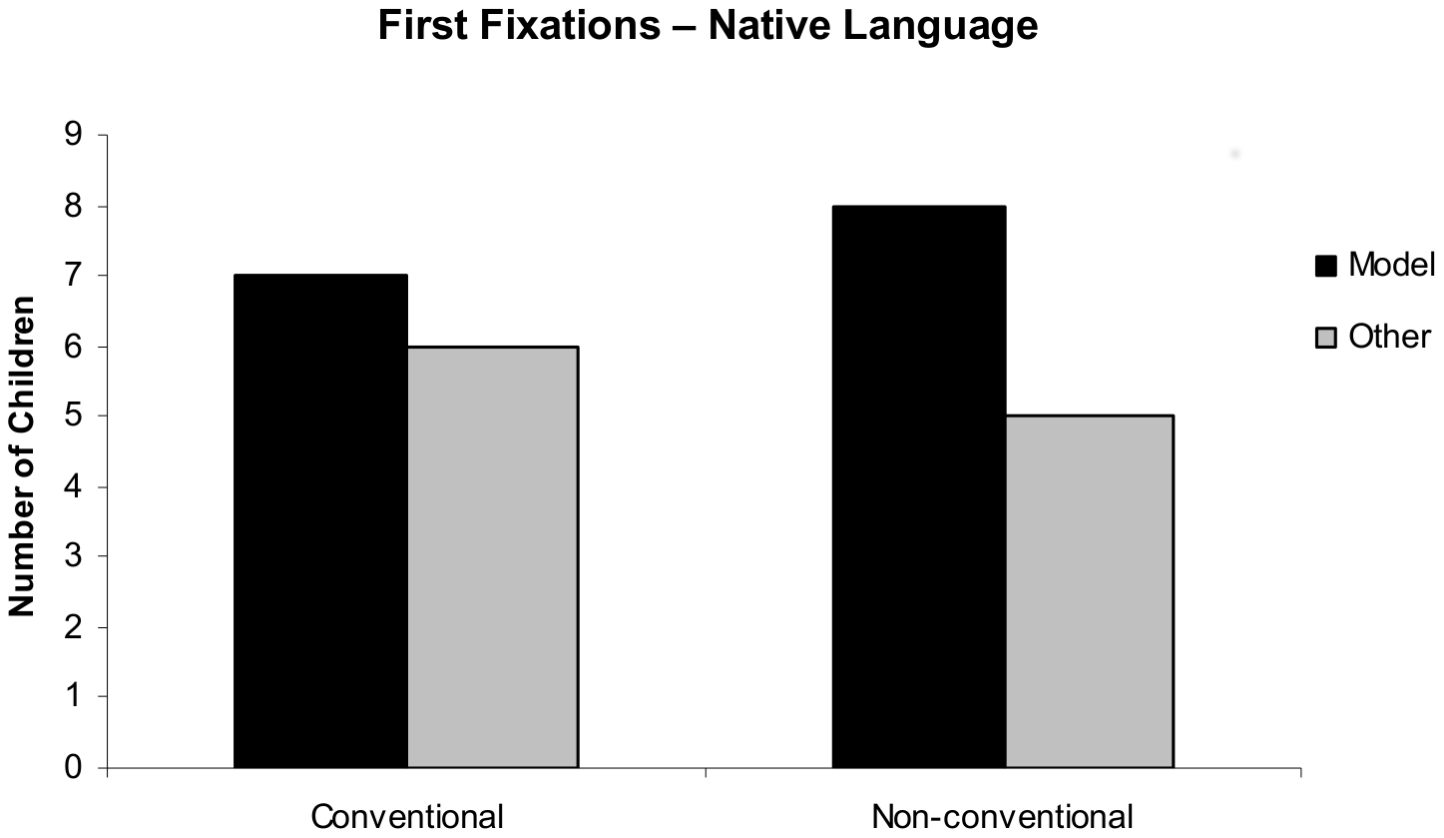
Distribution of first fixations after the onset of the audio stimulus in Experiment 2. Number of children looking first at the model and at the other person in the Conventional and the Non-conventional condition after hearing the native language text.

Analyzing the direction of the first fixations we found an effect of the experimental condition in the *before* (χ^2^ = 3.85, *p* = 0.05) time window showing that most of the children in the Conventional condition fixated on the model first (9 out of 13 participants) while the majority of children in the Non-conventional condition fixated on the other person first (9 out of 13 participants). However, *after* the onset of the stimulus there was no difference between conditions (*χ^2^* = 0.16, *p* = 0.69) with approximately the same number of children looking at the model first in both conditions (7 and 8 in the Conventional and Non-conventional condition, respectively). Results are depicted in Figure 4.

### Discussion

In this experiment we investigated whether 2-year-old children form similar representations of a person based on the observed level of conventionality in their tool using habits as they do based on the language they speak. This design allowed us to test whether children would associate a foreign and a native language differentially to people based on the conventionality of his behavior. We found that children associated a foreign language to the model if he had previously performed goal-directed actions in a non-conventional way, but formed an association between the foreign language and the other person if previously the model had been seen to act in a conventional way, making it unlikely that he was the source of the foreign language utterance. On the other hand, we found no evidence of children differentially associating a native language to the two men. The latter result indicates that different characteristics along which we form judgments about a person (or possibly about social group membership) may be organized hierarchically, and language represents a stronger cue than certain other qualities (in this case tool-use, but see [21]). Children meet a vast number of people every day that differ along countless traits but with a few exceptions they share a commonly spoken tongue. Therefore hearing a text in their native language will come as no surprise to them and will probably not elicit such a strong response from children. Supposedly this is reflected in the fact that we found no differences in Experiment 2.

First fixations were chosen as the subject of analyses as an indication of participants' expectation about who is more likely to be the source of the foreign language. Since children were presented with photographs, the question of to whom the voice belongs is rather ambiguous, which becomes evident to participants once they start to visually explore the static stimuli. However, the direction of the first fixation provides information about children's expectations before the realization that the visual stimuli will not help clear the ambiguity.

Analyzing the total visit durations in the test phase, we found a preference towards the model before the onset of the audio stimulus. This suggests that familiarity did have an effect in the test phase, but preferences based on mere familiarity faded away by the time the audio stimulus started and were also suppressed by the conflicting information that was provided about the model's behavioral habits (this is reflected in the between-conditions difference in first fixations in Experiment 1 and the even distribution of first fixations in Experiment 2).

Our results suggest that children take the familiarity and the conventionality of performed actions into account in forming representations about a person, and they generalize to other qualities (in this case, language) of the person based on this information. The phenomenon that even young children organize information about people systematically has been recently shown in a study where 6-month-old infants matched a non-native language to an other-race face [31]. This study, similarly to ours analyzed the looking-time patterns with photographs and spoken texts as stimuli.

Spoken language has been shown to occupy a prominent role in children's representations of humans. Children not only prefer people belonging to the same linguistic group as themselves [18], but they extend this preference to objects associated with a linguistic in-group [20], and they selectively learn from people speaking their own language [19], [32]. This study has demonstrated that these representations are not constrained to the domain of language but are possibly part of a wider module designated to reason about humans in terms of familiarity and conventionality of their behavior. These characteristics are ultimately indications of whether a person is in possession of the same cultural knowledge as oneself.

Differentiating between people based on whether they share the same cultural knowledge has great adaptive value in managing every day life. In conducting interactions with others we unconsciously rely on an immense amount of shared knowledge without which our interactions would be infinitely more difficult, unsuccessful or even dangerous (think of the example of driving on the inappropriate side of the road).

From a developmental point of view, this differentiation also gains great importance as children are in the process of acquiring the necessary cultural knowledge to become a competent member of a particular society. For this, they must be able to identify reliable sources information, whose knowledge is likely to prove useful to them as well (see [20]). It is also worth noticing that children already at the age of two are extremely sensitive to violations of norms [33], which means that they are equipped with the ability to form judgments of violating or adhering to social conventions.

In sum, this study has demonstrated that young children rely on the familiarity of tool using actions in forming judgments about a person and these representations are convergent with the ones based on language use. We propose that these two characteristics are alike in that they both belong to the body of culturally accumulated and shared knowledge. This study opens the ground for further investigations aiming to test the potential role that cues of culturally shared knowledge play in representing social groups.

## Appendix

### Detailed description of the video stimuli

#### 1. Food vs. Hair

The setup includes a fork and a brush (tools), a plate of food and the model's hair being messy (implication of goals). In the Conventional condition the model uses the fork to eat some potato from a plate, while in the Non-conventional condition the model uses the fork to brush his hair.

#### 2. Liquid vs. Locket

The setup includes a key and a spoon (tools), a glass of liquid and a locket (implications of goals). In the Conventional condition the model uses the key to open the locket, while in the Non-conventional condition the model uses the key to stir the liquid.

#### 3. Banana vs. Paper

The setup includes a knife and a pair of scissors (tools), a banana and some crepe paper (implications of goals). In the Conventional condition the model uses the scissors to cut the paper, while in the Non-conventional condition the model uses them to cut a banana.
